# A novel deep-benthic sea cucumber species of *Benthodytes* (Holothuroidea, Elasipodida, Psychropotidae) and its comprehensive mitochondrial genome sequencing and evolutionary analysis

**DOI:** 10.1186/s12864-024-10607-5

**Published:** 2024-07-13

**Authors:** Yingying He, Hancheng Zhao, Yongxin Wang, Changfeng Qu, Xiangxing Gao, Jinlai Miao

**Affiliations:** 1 Marine Natural Products Research and Development Laboratory, First Institute of Oceanography, Ministry of Natural Resources, Qingdao, 266061 China; 2 Laboratory for Marine Drugs and Bioproducts, Qingdao Marine Science and Technology Center, Qingdao, 266237 China; 3 National Deep Sea Center, Qingdao, 266237 China; 4 College of Chemistry and Chemical Engineering, Qingdao University, Qingdao, 266071 China; 5 Marine Functional Food Technology Innovation Center of Shandong Province, Rongcheng, 264306 China

**Keywords:** *Benthodytes* sp. Gxx-2023, Sea cucumber, Mitogenome, Bioinformatics analysis, Phylogenetic evolution

## Abstract

**Background:**

The holothurians, commonly known as sea cucumbers, are marine organisms that possess significant dietary, nutritional, and medicinal value. However, the National Center for Biotechnology Information (NCBI) currently possesses only approximately 70 complete mitochondrial genome datasets of Holothurioidea, which poses limitations on conducting comprehensive research on their genetic resources and evolutionary patterns. In this study, a novel species of sea cucumber belonging to the genus *Benthodytes*, was discovered in the western Pacific Ocean. The genomic DNA of the novel sea cucumber was extracted, sequenced, assembled and subjected to thorough analysis.

**Results:**

The mtDNA of *Benthodytes* sp. Gxx-2023 (GenBank No. OR992091) exhibits a circular structure spanning 17,386 bp, comprising of 13 protein-coding genes (PCGs), 24 non-coding RNAs (2 rRNA genes and 22 tRNA genes), along with two putative control regions measuring 882 bp and 1153 bp, respectively. It exhibits a high AT% content and negative AT-skew, which distinguishing it from the majority of sea cucumbers in terms of environmental adaptability evolution. The mitochondrial gene homology between Gxx-2023 and other sea cucumbers is significantly low, with less than 91% similarity to *Benthodytes marianensis*, which exhibits the highest level of homology. Additionally, its homology with other sea cucumbers is below 80%. The mitogenome of this species exhibits a unique pattern in terms of start and stop codons, featuring only two types of start codons (ATG and ATT) and three types of stop codons including the incomplete T. Notably, the abundance of AT in the Second position of the codons surpasses that of the First and Third position. The gene arrangement of PCGs exhibits a relatively conserved pattern, while there exists substantial variability in tRNA. Evolutionary analysis revealed that it formed a distinct cluster with *B. marianensis* and exhibited relatively distant phylogenetic relationships with other sea cucumbers.

**Conclusions:**

These findings contribute to the taxonomic diversity of sea cucumbers in the Elasipodida order, thereby holding significant implications for the conservation of biological genetic resources, evolutionary advancements, and the exploration of novel sea cucumber resources.

**Supplementary Information:**

The online version contains supplementary material available at 10.1186/s12864-024-10607-5.

## Background

Deep sea, refers to the ocean depths exceeding 1000 m, characterized by extreme environmental conditions including extremely low temperatures (mean temperature < 4 °C), high hydrostatic pressure (mean pressure of 400 atm), and the largest hypoxic and anoxic environments on Earth. With an average depth of approximately 4.2 km, the absence of sunlight results in near-total darkness, leading to a loss of net photosynthetic primary productivity. Despite these challenging, the deep-sea environment harbors abundant marine biological resources, potentially encompassing up to 1.5 million undiscovered species [1]. Remarkably adaptable organisms inhabit this realm, thriving within a temperature range from − 2 °C to > 150 °C and even surviving in sediments at depths of 10,000 m [2]. Due to their adaptation to such extreme conditions, deep-sea species exhibit distinct genetic characteristics compared to those found in shallow seas and terrestrial environments.

Sea cucumbers, belonging to the phylum Echinodermata, exhibit high nutritional value and are found in both deep sea and shallow water environments. Their genetic and metabolic characteristics enable them to produce secondary metabolites with potent biological activities, such as saponins and chondroitin sulfate, etc., which possess significant nutritional and medicinal value. Consequently, sea cucumbers represent a valuable resource for the prevention and treatment of various human diseases [3]. However, due to excessive exploitation driven by consumer demand coupled with inadequate fisheries management practices in numerous regions, there has been a severe depletion of natural sea cucumber populations [4]. Intensified efforts must be directed towards protecting and managing these invaluable genetic resources to ensure their sustainable utilization. To safeguard the stability and diversity of sea cucumber populations, diverse types of germplasm resources are collected and conserved, while novel varieties exhibiting superior traits such as rapid growth, high yield, and robust stress resistance are developed through genetic enhancement and crossbreeding techniques to meet market demands and optimize breeding efficiency.

We conducted a comprehensive search on the latest National Center for Biotechnology Information (NCBI) database (https://www.ncbi.nlm.nih.gov/Taxonomy/Browser/wwwtax.cgi?mode=Undef&id=7705&lvl=3&lin=f&keep=1&srchmode=1&unlock), revealing a total of 918 sea cucumber species, out of which 309 remain unidentified. According to the most recent classification standards [5, 6], the remaining 609 species can be categorized into 125 genera, distributed among 22 families and seven orders. At the order level, Dendrochirotida encompasses 63 genera, while both Synallactida and Apodida each consist of 18 genera. Elasipodida comprises 14 genera, Molpadida contains six genera, Holothuriida includes five genera, and Persicalida consists of one genus. The distribution proportions across different sea cucumber species are as follows: Dendrochirotida (30.54%), Synallactida (20.69%), Molpadida (2.63%), Persicalida (0.16%), Holothuriida (20.85%), Elasipodida (10.84%), and Apodida (14.29%). At the family level, the top five in terms of proportion are Holothuriidae (20.85%), Cucumariidae (12.48%), Stichopodidae (12.32%), Phyllophoridae (8.70%), Synaptidae (7.88%). Among the identified 125 genera, *Holothuria* exhibits the highest diversity with approximately 13.79% of all recorded sea cucumber species. The genus *Stichopus* accounts for 8.37%, making it the second most diverse taxonomic group. The remaining genera have relatively lower proportions below 4%. In the past, a significant number of sea cucumber species and genetic resources remained largely unexplored and unanalyzed. With the development of exploration technology and molecular biological sequencing technology, researchers have discovered many new sea cucumber species and gene genetic resources in recent years. For instance, *Apostichopus japonicus* [7, 8], *Paelopatides* sp. Yap [9], *Stichopus horrens* [10], *Benthodytes marianensis* [11], *Euthyonidiella zulfigaris* sp. nov. and *Acaudina spinifera* sp. nov. [12]. Mitochondria, serving as the primary sites for cellular respiration and energy production, assume a pivotal role in cellular and organismal growth and development [13]. Notably, mitochondrial DNA (mtDNA), despite constituting less than 1% of eukaryotic total DNA, offers unique advantages over nuclear DNA for studying species origin, phylogeny, as well as genetic differentiation among related species and interspecific populations. mtDNA, along with its highly conserved regions such as *16S rRNA* and *cox1*, is frequently employed for organism identification, rendering it a more reliable tool for phylogenetic analysis. Consequently, mtDNA analysis has become not only a conventional method for genetic markers but also a research hotspot across various fields including evolutionary biology, genomics, and bioinformatics.

As of December 18, 2023, NCBI has cataloged numerous species of sea cucumbers; however, only 78 mitochondrial genomes have been documented, representing 6 orders and 10 families. Among these, the order Synallactida exhibits the highest species diversity, accounting for nearly fifty percent of the known mitochondrial genomes. To our knowledge, the presence of mitogenomes in Elasipodida is currently limited, particularly considering that in family Psychropotidae, only one species (*B. marianensis*) has been identified, excluding this work. This limitation has significantly constrained the researches on the molecular evolution of sea cucumbers belonging to this particular family. Therefore, further exploration and genomic sequencing of additional species from family Psychropotidae, as well as other families, will undoubtedly advance comprehension of genetics and evolution in sea cucumbers. Moreover, it has the potential to provide valuable insights into the economic industry associated with these organisms.

We conducted a comprehensive analysis of mitochondrial genome data from sea cucumbers deposited in the NCBI database to elucidate the distribution and classification of existing mitogenomes within this taxonomic group. Furthermore, we identified a novel sea cucumber species of *Benthodytes* sp. Gxx-2023 in the Western Pacific and performed extensive sequencing and analysis of its mitogenome, encompassing gene composition, codon usage bias, gene rearrangement patterns, as well as phylogenetic evolution. The objective was to unravel the distinctive characteristics of its mitogenome, ascertain the homology of genes and their encoded proteins, comprehend the functions and structural features of the encoded proteins, and unveil evolutionary trends among others. In brief, this study provides a solid foundation for understanding genetic evolution and facilitating exploration and utilization of sea cucumbers resources.

## Materials and methods

### Materials and sources

**Fig. 1:**
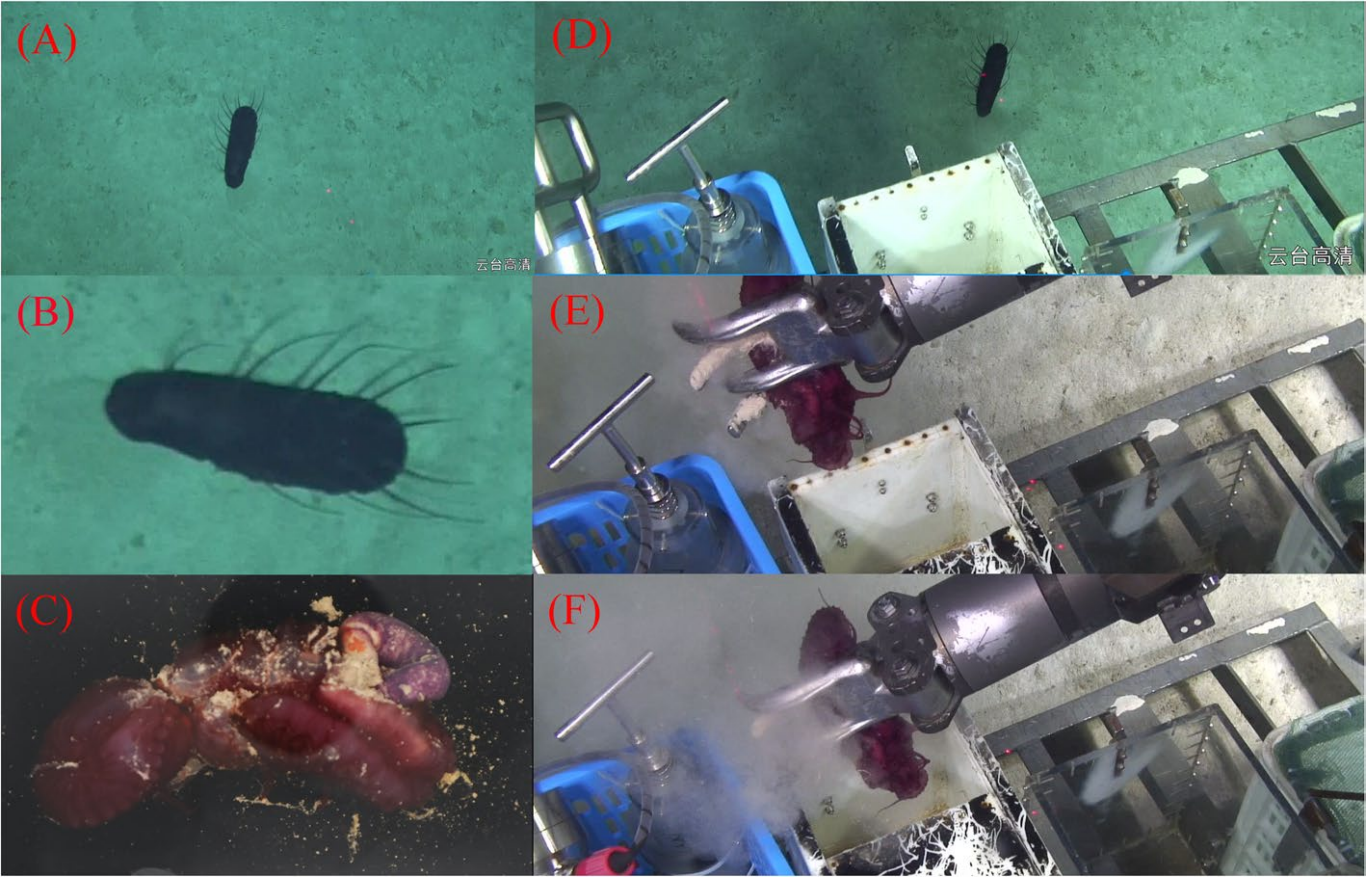
The characteristic and captured course of *Benthodytes* sp. Gxx-2023. **A** sea cucumber in situ, **B** zoomed-in sea cucumber in situ, **C** sea cucumber out of the seawater, **D**-**F** sea cucumber catching process

The benthic species *Benthodytes* sp. Gxx-2023 was captured on 17th-July-2023, at a depth of 2,446 m in the Western Pacific (15°30′26″N, 155°13′41″E) and was associated with China's first manned submersible support mother ship known as "Shenhai-1" (Fig. 1).

### mtDNA extraction and sequencing

DNA extraction from the sea cucumber *Benthodytes* sp. Gxx-2023 was performed using the DNeasy tissue kit (QIAGEN, Beijing, China). Following DNA isolation, approximately 1 μg of purified DNA was fragmented to an average size of 500 bp utilizing Covaris M220. Short-insert libraries were subsequently constructed according to the manufacturer's instructions (TruSeq™ Nano DNA Sample Prep Kit, Illumina) and subjected to sequencing on an Illumina NovaSeq 6000 platform (BIOZERON Co., Ltd., Shanghai, China), generating paired-end reads with a length of 150 bp.

### Mitogenome data preprocessing and assembly methods

Data processing involved trimming and filtering steps using Trimmomatic v0.39 software (http://www.usadellab.org/cms/index.php?page=trimmomatic) as follows [14]: i) removal of adapter sequences; ii) elimination of bases containing non-AGCT at the 5' end; iii) trimming low-quality ends based on a sequencing quality value threshold below Q20; iv) removal of reads containing more than 10% ambiguous bases represented by 'N'; v) discarding fragments shorter than 75 bp after adapter and quality pruning.

The mitochondrial genome assembly was performed using GetOrganelle v1.7.5 software (https://github.com/Kinggerm/GetOrganelle) [15]. Briefly, the target reads were retrieved using a seed database, followed by genome assembly using the SPAdes algorithm. Candidate sequences with high coverage depth and long assembly length were selected and confirmed as mitochondrial scaffolds through alignment to the NT library. These sequences were then connected based on overlap, and the starting position and orientation of the mitochondrial assembly sequence were determined by referencing the *B. marianensis* genome (accession number MH208310). Following these meticulous steps, we successfully obtained the mitogenome of *Benthodytes* sp. Gxx-2023.

### Mitochondrial genome structure annotation

The MITOS software (https://usegalaxy.eu/root?tool_id=toolshed.g2.bx.psu.edu%2Frepos%2Fiuc%2Fmitos2%2Fmitos2%2F2.1.3%20galaxy0) was utilized for the prediction of protein coding, tRNA, and rRNA genes within Gxx-2023 mitochondrial genome. Additionally, protein-coding genes (PCGs) were translated to amino acids according to non-vertebrate codon table. To ensure accuracy, any redundancy in the initial gene predictions from MITOS was eliminated, and manual corrections were made to determine precise start and end codon positions (SnapGene Viewer), resulting in a refined gene set with exceptional reliability. The complete and circular mitogenome of Gxx-2023 was visualized using CGView software (http://stothard.afns.ualberta.ca/cgview_server/). The functional annotations were conducted using Blast against publicly available protein databases, like NCBI non-redundant (Nr) protein database, Swiss-Prot, Clusters of Orthologous Groups (COGs), as well as Kyoto Encyclopedia of Genes and Genomes (KEGG) and Gene Ontology (GO) terms, with a typical cut-off E-value of 10^–5^. AT-skew = (A-T)/(A + T); GC-skew = (G-C)/(G + C) [16]. The homology analysis of the genes and their encoded proteins was conducted using blastn (https://blast.ncbi.nlm.nih.gov/Blast.cgi?PROGRAM=blastn&PAGE_TYPE=BlastSearch&BLAST_SPEC=&LINK_LOC=blasttab&LAST_PAGE=blastp) with an algorithm of highly similar sequences (megablast), and blastp (https://blast.ncbi.nlm.nih.gov/Blast.cgi?PROGRAM=blastp&PAGE_TYPE=BlastSearch&BLAST_SPEC=&LINK_LOC=blasttab&LAST_PAGE=blastp) with an algorithm of blastp (protein–protein BLAST) respectively. The remaining algorithm parameters are all set to their default values. The protein domains of the 13 PCGs were also analyzed using blastp.

### Analysis of Relative synonymous codon usage (RSCU)

The relative probability of a specific codon in the synonymous codon encoding the corresponding amino acid can serve as an indicator of codon usage preference. We determined the preference value for *Benthodytes* sp. Gxx-2023 codons by calculating RSCU using the “cusp” of EMBOSS package (v6.6.0.0) with in-house python [17].

### Comparative and rearrangement analyses of genome structures

The selected mitochondrial genome ring sequences were arranged linearly based on the *cox1* as the starting point, enabling analysis of gene sequence and rearrangement changes.

### Phylogenetic analysis

The mitogenome sequences of the sea cucumbers, as well as *Ophiura kinbergi* (MH910618), as an outgroup, were retrieved from NCBI database. Subsequently, the phylogenetic evolutionary tree was constructed using 13 conserved coding genes shared among the species. These coding gene sequences were aligned with MUSCLE v3.8.31 software using the codon table of 9, trimming the aligned regions with Gblocks v0.91b to obtain conserved regions, and determining the best nucleotide substitution model via jModeltest v2.1.10 software. Maximum likelihood (ML) estimation was performed on both the evolutionary tree and model parameters, where AIC and BIC scores were utilized to identify the optimal model. The obtained gene nucleotide sequences were found to adhere to an optimal GTR + I + G model. Finally, PhyML v3.0 (https://github.com/stephaneguindon/phyml) was employed for constructing a ML phylogenetic tree utilizing the GTR + I + G model [18].

## Results

### Current status of sea cucumber mitogenome

**Fig. 2:**
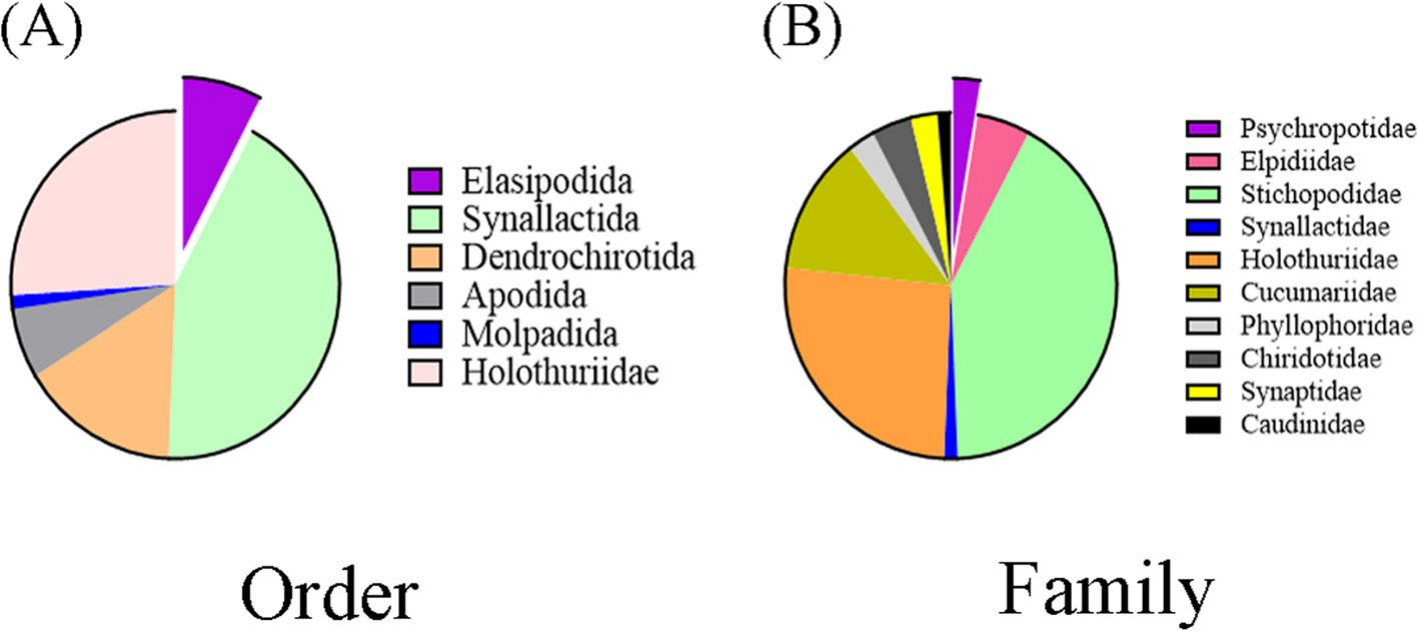
Distribution and abundance maps of orders (**A**) and families (**B**) with known mitogenomes in sea cucumbers. The purple color highlights the order and family to which *Benthodytes* sp. Gxx-2023 belongs

We retrieved the mitochondrial genome sequences of all sea cucumber species, including unidentified and incomplete ones, from the NCBI database up to December 18, 2023. Finally, A total of 78 mitogenomes were downloaded, excluding duplicate submissions. The taxonomic distribution of these sea cucumbers at order and family levels is presented in Fig. 2A and Fig. 2B, while summary Table S1 provides information on their mitochondrial genomes such as size and GenBank number. Among the sequenced mitochondrial genomes, 70 are complete while the remaining eight are partial sequences. *B. marianensis* possesses the largest complete mitochondrial genome, measuring 17,567 bp, while *Benthodytes* sp. Gxx-2023 ranks fourth with a total length of 17,386 bp. *Peniagone* sp. YYH-2013 possesses the smallest complete mitogenome with a length of 15,507 bp.

Except for one unidentified species of sea cucumber (*Holothuroidea* sp. FZ-2017, MF667551.1), the remaining 77 sea cucumbers are classified into six orders: Elasipodida, Dendrochirotida, Apodida, Molpadida, Synallactida, and Holothuriida, encompassing 10 families (Psychropotidae, Elpidiidae, Stichopodidae, Synallactidae, Holothuriidae, Cucumariidae, Phyllophoridae, Chiridotidae, Synaptidae, and Caudinidae) along with 25 genera (*Benthodytes*, *Peniagone*, *Scotoplanes*, *Parastichopus*, *Apostichopus*, *Isostichopus*, *Stichopus*, *Thelenota*, *Synallactes*, *Holothuria*, *Bohadschia*, *Actinopyga*, *Cucumaria*, *Colochirus*, *Thyonella*, *Ocnus*, *Cercodemas*, *Neocucumis*, *Pseudocolochirus*, *Phyllophorella*, *Phyrella*, *Chiridota*, *Protankyra*, *Euapta*, *Acaudina*).

At the order level, Synallactida had the largest number of sea cucumbers, with 33 species, accounting for 42.31%, followed by Holothuriida and Dendrochirotida, accounting for 25.64% and 15.38%, and Molpadida had the smallest number, with only one sea cucumber mitochondrial genome sequenced and published (Fig. 2A). At the family level, Stichopodidae, Holothuriidae and Cucumariidae are the most common families of sea cucumber, accounting for 41.03%, 25.64% and 12.82%, respectively (Fig. 2B). Among them, *Apostichopus*, *Holothuria* and *Stichopusssh* accounted for 24.36%, 20.51% and 11.54% respectively, accounting for more than half of the total. The family Psychropotidae, to which *Benthodytes* sp. Gxx-2023 belongs, comprises only two species, including itself of course.

### Whole mitogenome information of Gxx-2023

**Fig. 3:**
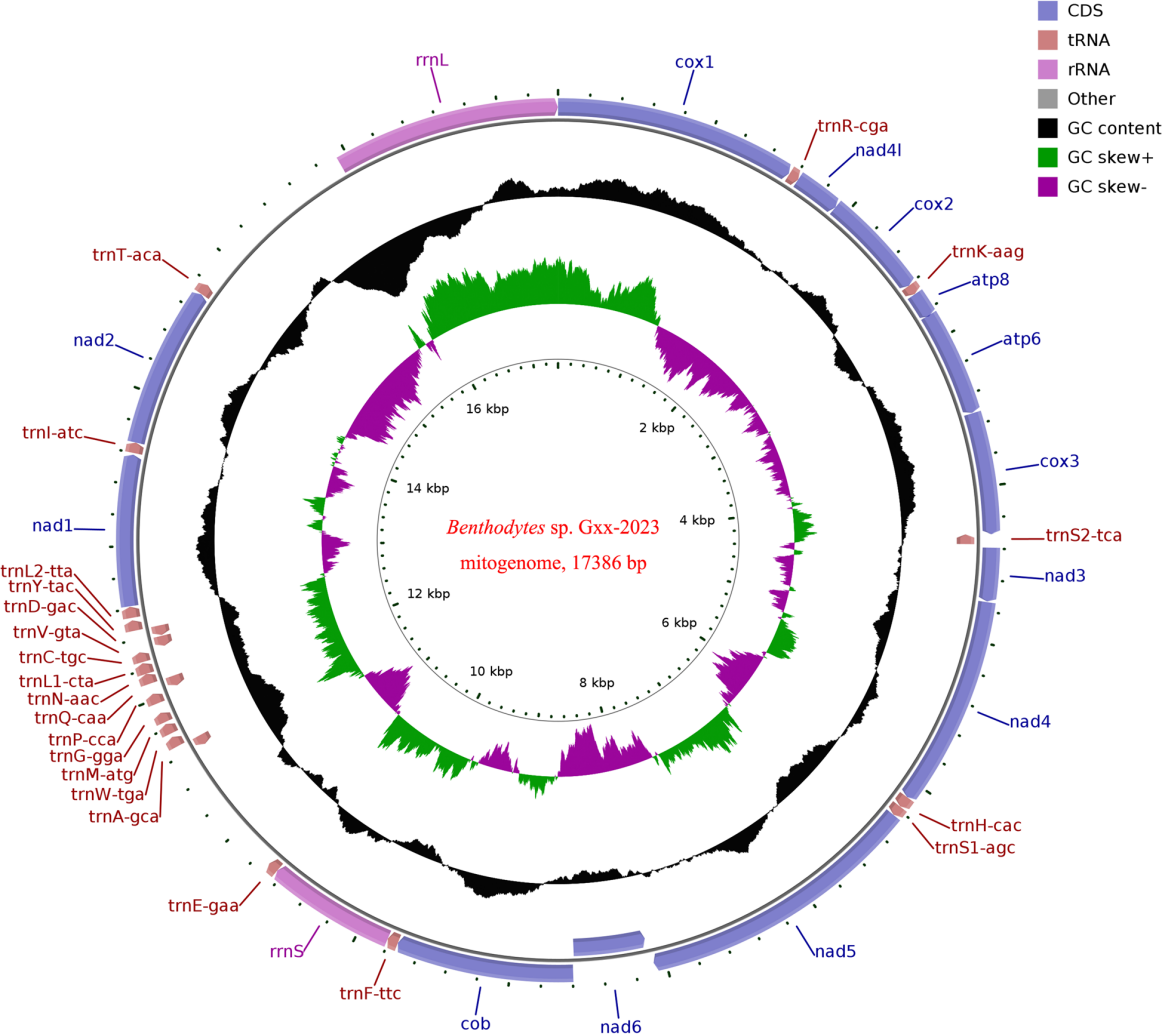
The complete mitochondrial genome of *Benthodytes* sp. Gxx-2023

The insert size of the mitochondrial genome in the next generation sequencing is 450 bp, with raw data and clean data sizes of 7,761.6 Mb and 7,543.9 Mb respectively. The Q20 values for the clean data are at 99.04%, while the Q30 values stand at 97.33%. Additionally, the GC content is measured to be 41.71%. The sequencing depth and coverage information (the average depth was 110.91 × and the coverage was 100%) of *Benthodytes* sp. Gxx-2023 are presented in Figure S1, demonstrating high-quality mitogenomic data. After conducting sequencing, assembly, and annotation analysis, the complete mitochondrial genome data of *Benthodytes* sp. Gxx-2023 (GenBank No. OR992091) has been acquired, as depicted in Fig. 3. It exhibits a circular structure with a sequence size of 17,386 bp and encompasses 13 PCGs, 24 non-coding RNAs (including 2 rRNA genes and 22 tRNA genes), along with two putative control regions measuring 882 bp and 1,153 bp respectively.

The genome base composition and gene distribution of *Benthodytes* sp. Gxx-2023 were statistically analyzed and summarized (Table 1). It was observed that the base composition consisted of T (36.51%), C (18.70%), A (31.35%), and G (13.44%). The AT content was calculated to be 65.33%. Furthermore, the AT contents in tRNA, rRNA and PCGs were determined to be 66.11%, 70.37%, and 68.77% respectively, while the mitogenome exhibited an AT-skew of -0.07603 and a GC-skew of -0.16366. The first, second, and third bases of all triplet codons within the 13 PCGs had AT contents of 65.43%, 66.56%, and 66.35% respectively.

**Table 1:** Nucleotide composition and AT-GC skewness of the *Benthodytes* sp. Gxx-2023 mitogenome

*Benthodytes* sp. Gxx-2023	number	Length (bp)	Gene length/Genome (%)	T%	C%	A%	G%	AT%	GC%	AT-Skew	GC-Skew
Genome	1	17,386	100	36.51	18.70	31.35	13.44	67.86	32.14	-0.07603	-0.16366
PCGs	13	11,358	65.33	36.95	19.70	29.16	14.19	66.11	33.89	-0.11783	-0.16258
First position^a^	3,786	3,786	21.78	35.60	20.52	29.82	14.05	65.43	34.57	-0.08835	-0.18716
Second position^a^	3,786	3,786	21.78	38.51	19.28	28.05	14.16	66.56	33.44	-0.15715	-0.15311
Third position^a^	3,786	3,786	21.78	36.74	19.28	29.61	14.37	66.35	33.65	-0.10746	-0.14591
tRNA	22	1,505	8.66	35.28	14.22	35.08	15.42	70.37	29.63	-0.00284	0.04049
rRNA	2	2,283	13.13	33.25	15.72	35.52	15.51	68.77	31.23	0.033009	-0.00672
Control region	2	2,035	11.70	42.31	16.86	30.96	9.88	73.27	26.73	-0.15491	-0.26103

^a^First position, Second position and Third position respectively present the first, second and third bases of all triplet codons of PCGs

### mtDNA composition, distribution and homology analysis

The mitochondrial genome of *Benthodytes* sp. Gxx-2023 comprises a total of 37 genes, including 13 protein-coding genes, 22 tRNA genes, and 2 rRNA genes. The 13 PCGs are, namely, *cytochrome c oxidase subunit 1* (*cox1*), *cytochrome c oxidase subunit 2* (*cox2*), and *cytochrome c oxidase subunit 3* (*cox3*), *cytochrome b* (*cytb*), *ATP synthase F0 subunit 6* (*atp6*), *ATP synthase F0 subunit 8* (*atp8*). *NADH dehydrogenase subunit 1* (*nad1*), *NADH dehydrogenase subunit 2* (*nad2*), *NADH dehydrogenase subunit 3* (*nad3*), *NADH dehydrogenase subunit 4* (*nad4*), *NADH dehydrogenase subunit 5* (*nad5*), *NADH dehydrogenase subunit 6* (*nad6*), *NADH-ubiquinone/plastoquinone oxidoreductase, chain 4l* (*nad4l*). Notably, *nad6* is located on the R chain whereas the remaining twelve are situated on the F chain. Of the twenty-two tRNA molecules identified, five were found to be located on the R chain (*trnS2-tca*, *trnD-gac*, *trnV-gta*, *trnQ-caa* and *trnA-gca*) while the others were located on the F chain. Both rRNAs (*rrnS* and *rrnL*) are exclusively situated on the F strand.

The Blast analysis of the mitogenome genes of Gxx-2023 revealed relatively low homology with other sea cucumber mitochondrial genes, and exhibited an average homology of only 90.97% with *B. marianensis* (Table S2). The homology between *cox1* sequences from Gxx-2023 and *B. marianensis* was 91.89%, while the similarity between *Stichopus chloronotus* and *Stichopus chloronotus* strain lv was found to be 78.52%; The *cox2* of Gxx-2023 had the highest homology of 91.86% with that of *B. marianensis*, followed by *Thyonella gemmate* with only 77.86%; The *cox3* of Gxx-2023 had the highest homology of 92.85% with that of *B. marianensis*, followed by *Cucumaria frondosa* with only 76.53%. The *nad1* of Gxx-2023 exhibited the highest sequence similarity of 89.92% with that of *B. marianensis*, followed by *Phyllophorella liuwutiensis* with a comparatively lower homology of only 73.17%; The *nad2* of Gxx-2023 exhibited the highest sequence similarity of 86.55% with that of *B. marianensis*, followed by *Peniagone* sp. YYH-2013 with a comparatively lower homology of only 68.16%; The *nad3* gene of Gxx-2023 exhibited the highest sequence similarity of 90.43% with that of *B. marianensis*, followed by *P. liuwutiensis* with a comparatively lower homology of only 74.49%; The *nad4* of Gxx-2023 exhibited the highest sequence similarity of 88.68% with that of *B. marianensis*, followed by *Scotoplanes* sp. H8 with a comparatively lower homology of only 69.76%; The *nad5* of Gxx-2023 exhibited the highest sequence similarity of 90.67% with that of *B. marianensis*, followed by *Peniagone* sp. YYH-2013 with a comparatively lower homology of only 71.75%; The *nad6* of Gxx-2023 exhibited the highest sequence similarity of 92.42% with that of *B. marianensis*, followed by *Cercodemas anceps* with a comparatively lower homology of only 74.17%; The *nad4l* gene of Gxx-2023 exhibited the highest sequence similarity of 91.58% with that of *B. marianensis*, followed by *S. chloronotus* and *S. chloronotus* strain lv with a comparatively lower homology of only 75.19%. The *cytb* sequence of Gxx-2023 exhibited the highest degree of homology (90.56%) with that of *B. marianensis*, followed by *Scotoplanes* sp. H8 with a comparatively lower homology level (73.68%); The *atp6* sequence of Gxx-2023 exhibited the highest degree of homology (92.4%) with that of *B. marianensis*, followed by *Scotoplanes* sp. H8 and *Scotoplanes* sp. H5 with a comparatively lower homology level (71.57%); It is noteworthy that *atp8* exhibits the highest homology of 92.86% with *B. marianensis*, while displaying significantly lower homology with other sea cucumbers (almost no homology).

### Homology and domain analysis of mitochondrial genes encoding proteins

The protein exhibits a relatively low level of homology (Table S3). Specifically, it shares approximately 97% homology with *B. marianensis*, while displaying lower levels with other sea cucumbers. Additionally, the COX1 protein demonstrates the highest degree of similarity, exhibiting 91.1% homology with *Bohadschia argus*. Proteins such as NAD4L, ATP6, CYTB, COX2, and COX3 exhibit a range of 80%-90% homology. Proteins like NAD1, NAD3, and NAD4 display 70%-80% homology; whereas proteins like NAD2, NAD5, and NAD6 display homologies between 60 to 70%. On the other hand, ATP8 exhibits the lowest level of homology at only 52% with *Actinopyga lecanora* and less than 50% with other sea cucumbers.

**Fig. 4:**
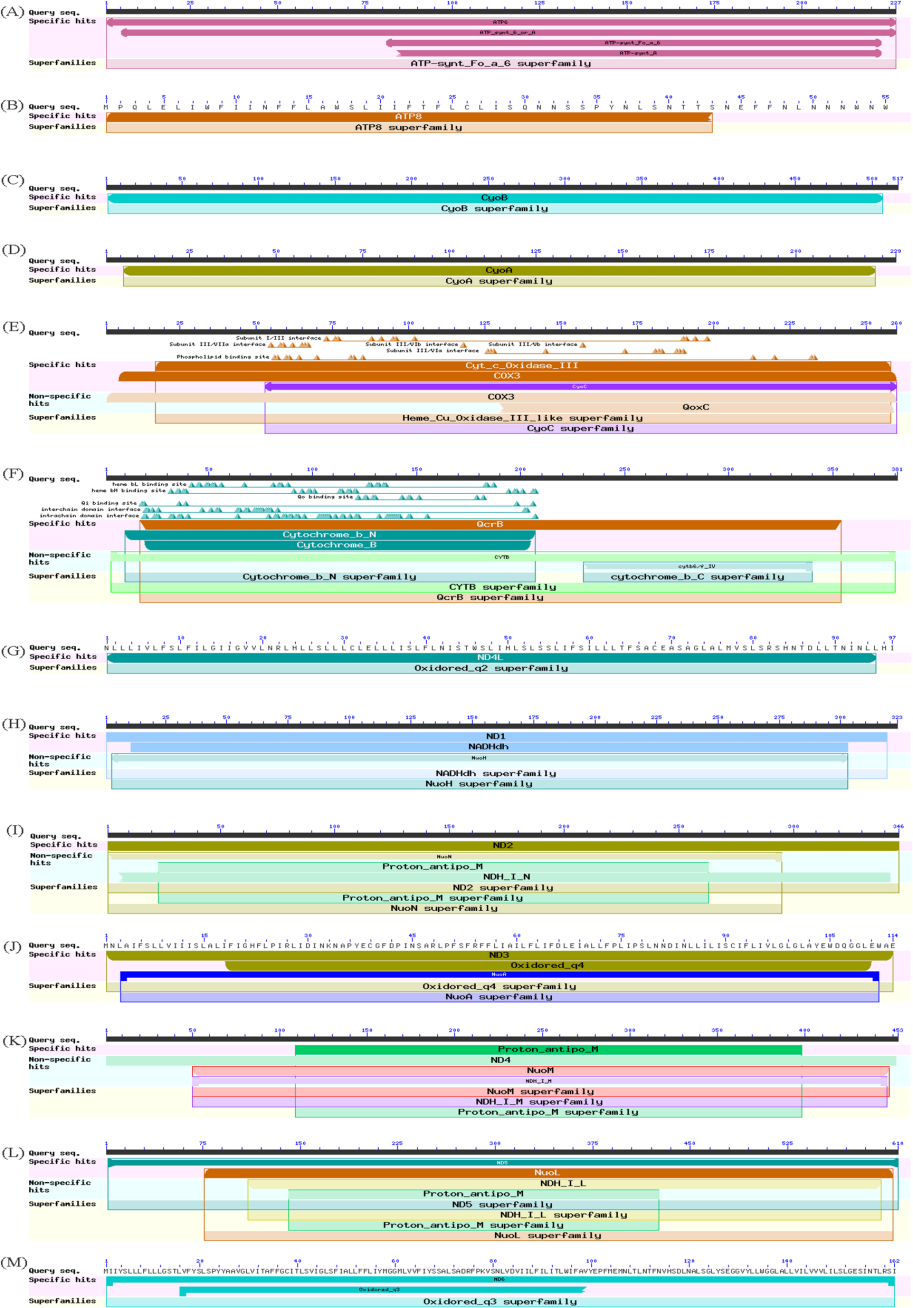
The protein domains encoded by mitochondrial genes in sea cucumbers. **A ***atp6*, **B** *atp8*, **C** *cox1*, **D** *cox2*, **E** *cox3*, **F** *cytb*, **G** *nad4l*, **H** *nad1*, **I** *nad2*, **J** *nad3*, **K** *nad4*, **L** *nad5*, **M*** nad6*

Among the mitochondrial proteins involved in energy production and conversion, the ATP8 subunit exclusively contains the ATP8 (MTH00036) domain, while the ATP6 subunit not only possesses the ATP6 (MTH00035) domain but also includes the ATP_synt_6_or_A (TIGR01131), ATP-synt_Fo_a_6 (cd00310), and ATP-synt_A (pfam00119) domains (Fig. 4A-B). The proteins COX1, COX2, and COX3 are members of the CyoB (COG0843), CyoA (COG1622), and CyoC (COG1845) superfamilies, respectively (Fig. 4C-E). Additionally, COX3 also belongs to the QoxC superfamily (TIGR02897). CYTB contains five domains namely CYTB (MTH00034), QcrB (COG1290), Cytochrome_b_N (cd00284), Cytochrome_B (pfam00033) and cytb6/f_IV (TIGR01156) (Fig. 4F). ND4L (MTH00043) is the only conserved domain found in NAD4L, while multiple domains are present in NAD1, NAD2, NAD3, NAD4 and NAD5 including ND1 (MTH00040), ND2 (MTH00041), ND3 (MTH00042), ND4 (MTH00044) and ND5 (MTH00208) respectively (Fig. 4G-M). Specifically, NADHdh (pfam00146) and NuoH (COG1005) domains are present in NAD1; NuoN (COG1007), Proton_antipo_M (pfam00361) and NDH_I_N (TIGR01770) domains are harbored by NAD2; Oxidored_q4 (pfam00507) and NuoA (COG0838) are encompassed by NAD3. Furthermore, NuoM (COG1008), NDH_I_M (TIGR01972) and Proton_antipo_M (pfam00361) differentiate from other genes for being incorporated into the structure of NAD4; whereas NDH_I_L (TIGR01974), NuoL (COG1009) and Proton_antipo_M (pfam00361) constitute for NAD5. Lastly, Oxidored_q3 (pfam00499) presents itself as a characteristic feature of NAD6.

**Fig. 5:**
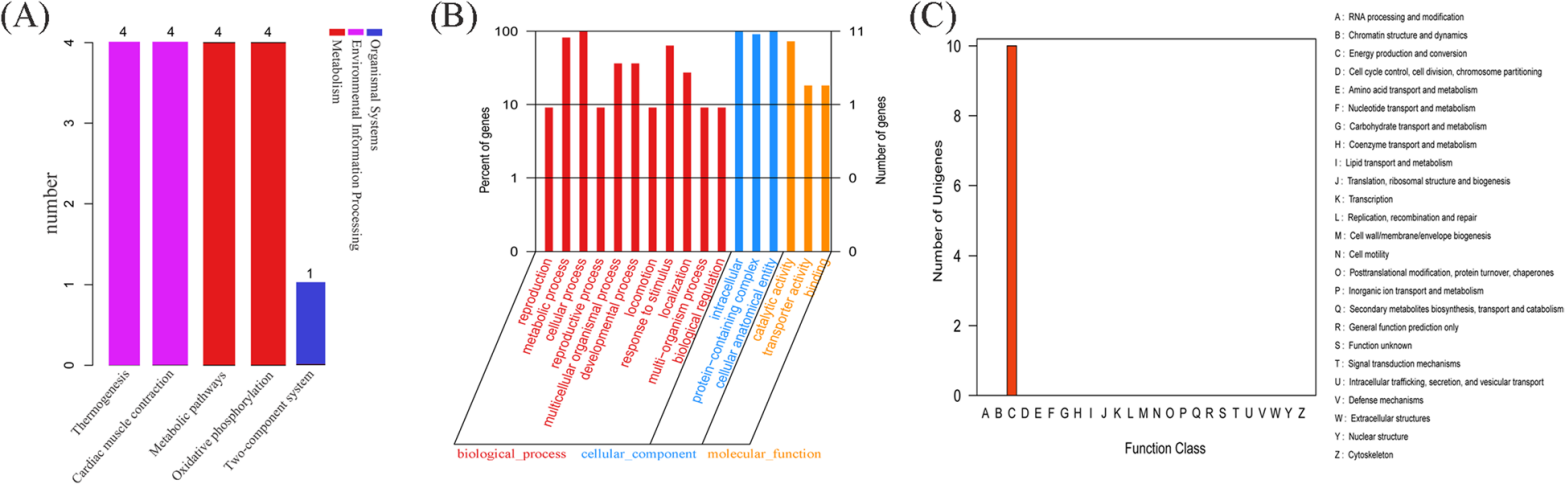
The functional annotations of mitochondrial genes in sea cucumbers. **A** KEGG, **B** GO, **C** eggNOG-KOG

KEGG analysis revealed that four genes (*cox1*, *cox2*, *cox3*, *cytb*) were associated with Oxidative phosphorylation and Metabolic pathways within the Metabolism category, as well as Organismal Systems and Thermogenesis pathways within the Organismal Systems category. Additionally, *cytb* was also found to be enriched in the Two-component system pathway under Environmental Information (Fig. 5A, Table S5). GO analysis indicated that 11 genes (*atp6*, *cytb*, *cox1*, *cox2*, *cox3*, *nad1*, *nad2*, *nad3*, *nad4*, *nad5*, *nad6*) were associated with three categories: biological processes (BP), cellular components (CC), and molecular functions (MF) (Table S4). Among these categories, BP exhibited the highest abundance with a total of eleven genes involved primarily in cellular process, metabolic processes, and response to stimulus (Fig. 5B, Table S5). EggNOG (KOG) analysis showed that ten genes (*cox1*, *nad2*, *nad4*, *cox3*, *nad3*, *atp6*, *cox2*, *nad5*, *nad1*, *cytb*) were significantly enriched in Energy production and conversion (Fig. 5C, Table S5).

### Gene intergenic spacer region and the overlapping region

The PCGs, rRNA, and tRNA of the mitochondrial genome of *Benthodytes* sp. Gxx-2023 are summarized in Table 2, presenting their gene sequence coordinates, gene length, gene interval length, codon usage, and other relevant information. A total of 22 spacer regions (2252 bp) are identified within the mitochondrial genome. Among these spacers, the largest one (1153 bp) is located between *trnT-aca* and *rrnL*. Notably, there were no gaps observed among the 18 genes; specifically, *trnR-cga*, *nad4l*, *cox2*, and *trnK-aag* form a continuous stretch while *cytb*, *trnF-ttc*, *rrnS*, and *trnE-gaa* also constitute another uninterrupted set of genes. Additionally, the mitochondrial genome contains three overlapping regions totaling to 13 bp; notably, the *atp8-atp6* overlap region is the longest with a size of 7 bp.

**Table 2:** *Benthodytes* sp. Gxx-2023 mitogenome genes and related information

NO	Gene	Strand	Position (Start–End)	Length (bp)	Intergenic spacer	Start codon	Stop codon
1	*cox1*	F	1–1,554	1,554	-	ATG	TAG
2	*trnR-cga*	F	1,560–1,625	66	5	-	-
3	*nad4l*	F	< 1,626–1,922	297	0	ATT	TAA
4	*cox2*	F	1,923–2,610	688	0	ATG	T
5	*trnK-aag*	F	2,611–2,675	65	0	-	-
6	*atp8*	F	2,677–2,844	168	1	ATG	TAA
7	*atp6*	F	2,838–3,521	684	-7	ATG	TAA
8	*cox3*	F	3,525–4,307	783	3	ATG	TAA
9	*trnS2-tca*	R	4,306–4,371	66	-2	-	-
10	*nad3*	F	4,394–4,738	345	22	ATG	TAA
11	*nad4*	F	4,743–6,102	1,360	4	ATG	T
12	*trnH-cac*	F	6,103–6,176	74	0	-	-
13	*trnS1-agc*	F	6,178–6,244	67	1	-	-
14	*nad5*	F	6,246–8,078	1833	1	ATG	TAA
15	*nad6*	R	8,101–8,589	489	22	ATG	TAA
16	*cytb*	F	8,598–9,741	1,144	8	ATG	T
17	*trnF-ttc*	F	9,742–9,811	70	0	-	-
18	*rrnS*	F	9,812–10,643	832	0	-	-
19	*trnE-gaa*	F	10,644–10,711	68	0	-	-
20	*trnA-gca*	R	11,594–11,661	68	882	-	-
21	*trnW-tga*	F	11,668–11,736	69	6	-	-
22	*trnM-atg*	F	11,760–11,828	69	23	-	-
23	*trnG-gga*	F	11,842–11,907	66	13	-	-
24	*trnP-cca*	F	11,971–12,038	68	63	-	-
25	*trnQ-caa*	R	12,035–12,104	70	-4	-	-
26	*trnN-aac*	F	12,106–12,172	67	1	-	-
27	*trnL1-cta*	F	12,173–12,244	72	0	-	-
28	*trnC-tgc*	F	12,252–12,316	65	7	-	-
29	*trnV-gta*	R	12,317–12,386	70	0	-	-
30	*trnD-gac*	R	12,392–12,458	67	5	-	-
31	*trnY-tac*	F	12,459–12,526	68	0	-	-
32	*trnL2-tta*	F	12,543–12,614	72	16	-	-
33	*nad1*	F	12,618–13,589	972	3	ATG	TAA
34	*trnI-atc*	F	13,601–13,668	68	11	-	-
35	*nad2*	F	13,669–14,709	1,041	0	ATG	TAA
36	*trnT-aca*	F	14,712–14,781	70	2	-	-
37	*rrnL*	F	15,935–17,385	1,451	1,153	-	-

### Codon and its usage bias

**Fig. 6:**
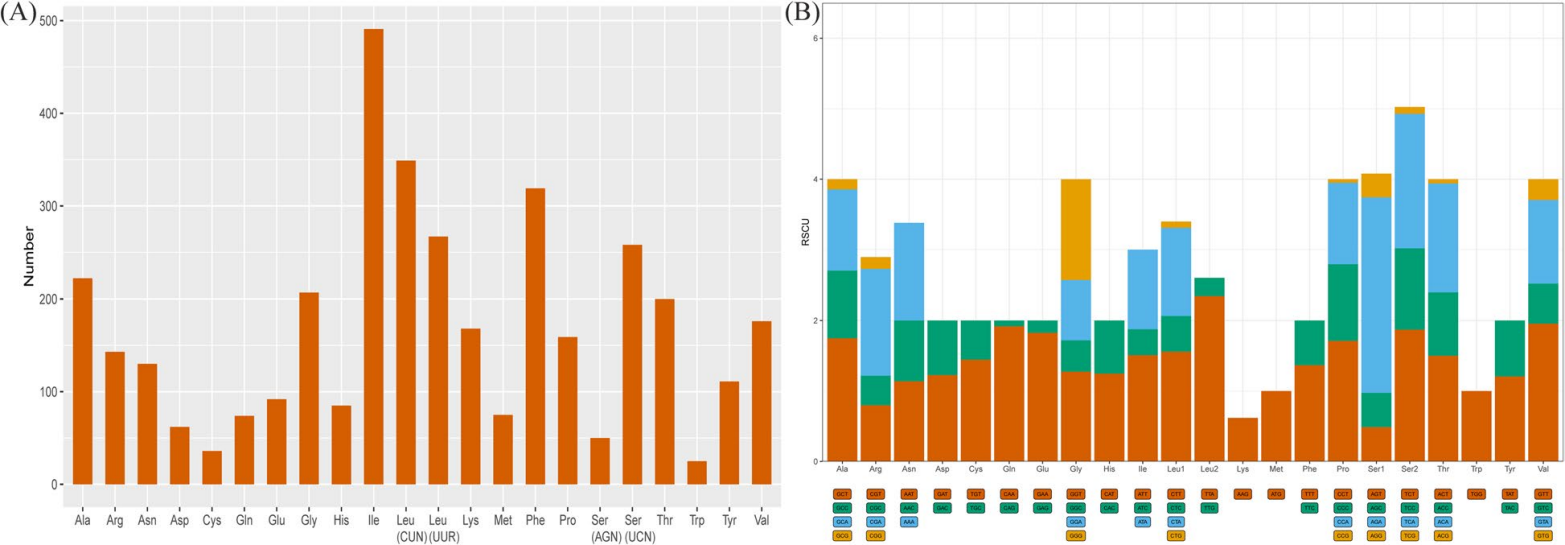
Codon number (**A**) and RSCU (**B**) of the mitogenome of *Benthodytes* sp. Gxx-2023. The horizontal coordinate represents the amino acids encoded by the codons in the mitogenome

The mitochondrial DNA of *Benthodytes* sp. Gxx-2023, as depicted in Fig. 6A, contains a total of 61 codons encoding 20 amino acids, apart from the stop codons. The codon Ile was found to be the most frequently utilized (491 occurrences), followed by Leu-CUN (349) and Phe (319), accounting for 13.27%, 9.43%, and 8.62% of the usage frequency, respectively. Conversely, Trp was identified as the least used codon with only 25 occurrences, followed by Cys (36) and Ser-AGN (50), representing a mere 0.68%, 0.97%, and 1.35%, respectively (Fig. 6A). Codons with an RSCU value exceeding 2 are arranged in descending order as AGA (Ser, 2.769), TTA (Leu, 2.347), GTT (Val, 1.955), CAA (Gln, 1.919), TCA (Ser, 1.909) (Fig. 6B).

The start codon ATG is utilized by all 12 genes in *Benthodytes* sp. Gxx-2023 mtDNA, except for *nad4l*, which employs ATT as the start codon (Table 2). Stop codons can be categorized into three types: TAA, TAG, and incomplete T. Among them, only *cox1* employs TAG as the stop codon, while *cox2*, *nad4*, and *cytb* use T as the stop codon. The majority of the remaining genes (a total of nine) employ TAA as their stop codon. Within these codons, there exist two distinct types of Leu and Ser: *trnL1-cta*, *trnL2-tta*, and *trnS1-agc*, *trnS2-tca*.

### Mitochondrial gene order and rearrangements

**Fig. 7:**
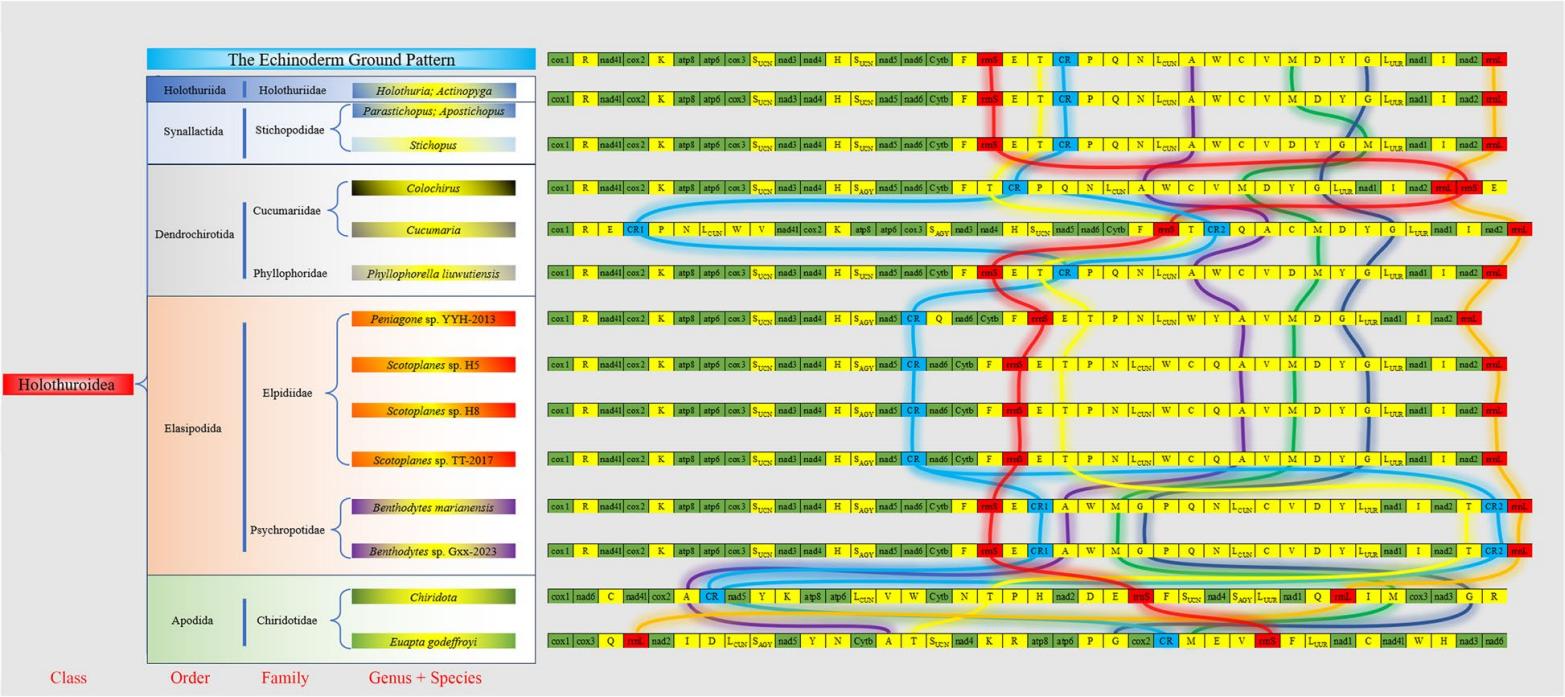
Mitochondrial gene rearrangements of *Benthodytes*

In the Fig. 7, we present a comprehensive list of sea cucumber species with measured mitochondrial genomes under Elasipodida, along with several representative sea cucumbers. The number and composition of PCGs in these sea cucumbers' mitochondrial genomes remain consistent. Except for *Benthodytes* sp. Gxx-2023, *B. marianensis*, and *Cucumaria* which have two control regions, all other sea cucumbers possess only one. Most sea cucumbers possess 22 tRNAs in their mtDNA; however, *Peniagone* sp. YYH-2013 (Elpidiidae), belonging to the same family as *Benthodytes* sp. Gxx-2023, only has 21 tRNAs. In terms of *rrnS* and *rrnL* rearrangements within the mtDNA structure, most species exhibit a conservative pattern with *rrnL* located towards the bottom; *Euapta godeffroyi* is an exception where it is positioned fourth.

With the exception of order Apodida and genus *Cucumaria* in order Dendrochirotida, the PCGs genes of sea cucumbers in other orders exhibited a relatively conserved arrangement (*cox1* → *nad4l* → *cox2* → *atp8* → *atp6* → *cox3* → *nad3* → *nad4* → *nad5* → *nad6* → *cytb* → *nad1* → *nad2*). Some tRNA genes showed rearrangements, particularly *tRNA-Thr*, *tRNA-Ala*, *tRNA-Met*, and *tRNA-Gly,* which are highlighted in yellow, purple, green, and dark blue respectively.

In Elasipodida, compared to the echinoderm ground pattern, sea cucumbers in Elpidiidae exhibits transpositions, such as *tRNA-Trp* genes and CR advancement; *rrnS*, *tRNA-Thr*, *tRNA-Ala*, etc., undergo retrograde movement. Notably, *tRNA-Thr* has been repositioned to the third position from the bottom accompanied by an additional CR at the penultimate position. In summary, the Psychropotidae group consisting of *Benthodytes* sp. Gxx-2023 and *B. marianensis* demonstrates extensive rearrangements.

### Phylogenetic relationships analysis

**Fig. 8:**
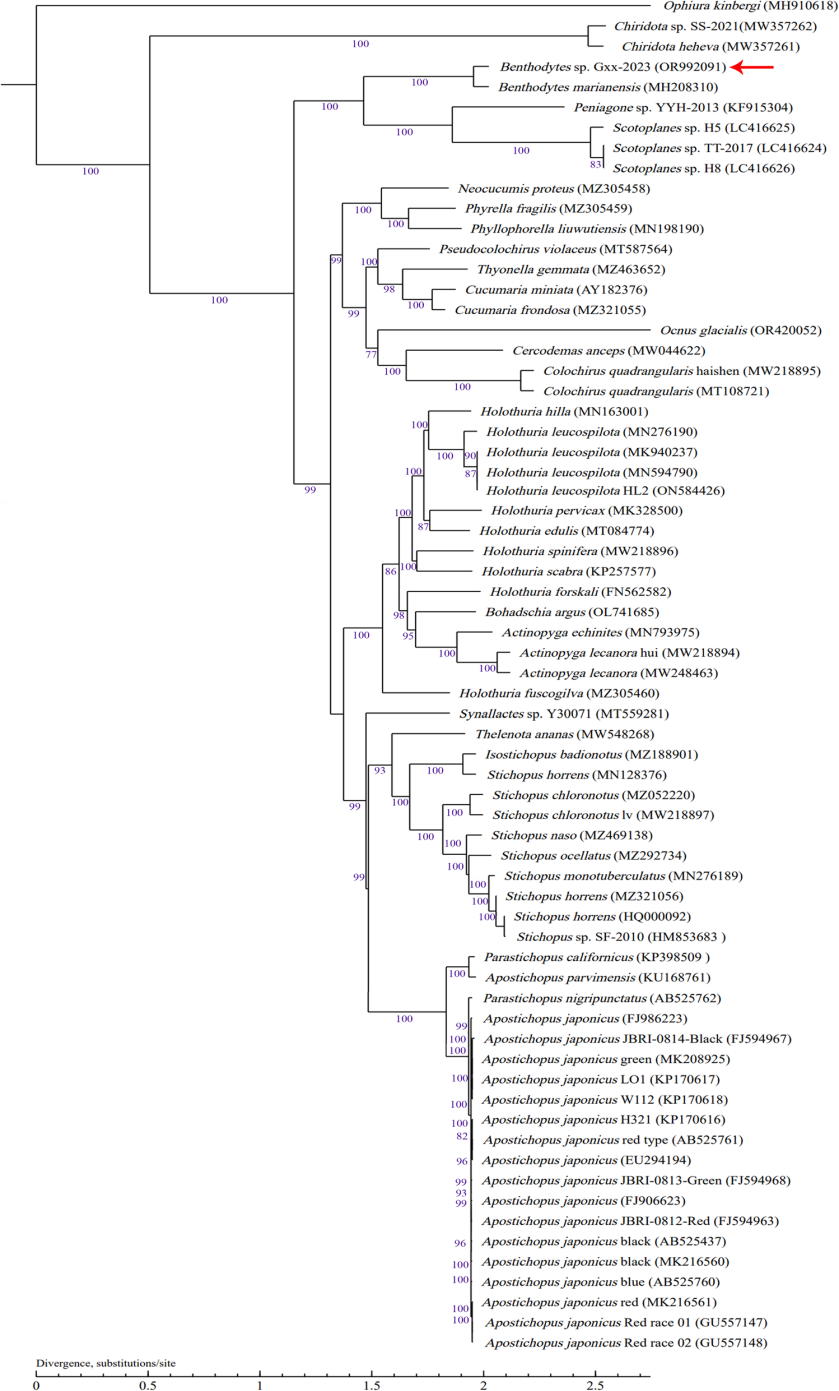
Phylogenetic relationships analysis of *Benthodytes* sp. Gxx-2023 mitogenome

Among the 78 sea cucumber mitochondria analyzed, we excluded 8 sea cucumbers lacking coding genes (*Apostichopus californicus* isolate PC1234 (CM036232), *Holothuria polii* (LR694133), *Holothuroidea* sp. FZ-2017 (MF667551), *Holothuria fuscocinerea* (MK391177), *Holothuria leucospilota* (MK801674), *Colochirus robustus* (MN966676), *Chiridotidae* sp. KJ-Belize-E1_1 (MT877116), *Protankyra verrilli* (ON018239)), as well as 3 sea cucumbers with incomplete gene prediction (*Acaudina molpadioides* (MK050109), *H. cocinerea* (MN542416), *Holothuria spinifera* strain S-4 (MN816440)). *Benthodytes* sp. Gxx-2023 belongs to Eukaryota, Metazoa, Echinodermata, Eleutherozoa, Echinozoa, Holothuroidea, Elasipodida, Psychropotidae, *Benthodytes*. By utilizing *O. kinbergi*, a species belonging to the family Ophiuridae, as an outgroup, the topology of the phylogenetic tree constructed based on 67 complete mitochondrial genomes of sea cucumbers exhibits substantial congruence with conventional taxonomic classifications (Fig. 8). The findings revealed that *Benthodytes* sp. Gxx-2023 belongs to the same evolutionary lineage as *B. marianensis* (accession number MH208310), and they exhibit a close phylogenetic relationship with members of the Elpidiidae family (accession numbers KF915304, LC416625, LC416624).

## Discussion

Although there exist thousands of sea cucumber species, only 70 complete mitochondrial genomes have been sequenced thus far. Notably, Elasipodida encompasses both the largest (*B. marianensis*) and smallest (*Peniagone* sp. YYH-2013) sea cucumbers in terms of mitogenome length. This finding is intriguing due to the limited availability of only five complete genomes, yet it encompasses the largest and smallest sea cucumbers within Holothurioidea. The collection of 78 mitochondrial genomes from sea cucumbers represents ten families across six orders: Elasipodida, Dendrochirotida, Apodida, Molpadida, Synallactida and Holothuriida. Among these orders, Synallactida is the most prevalent with a representation of 42.31%, followed by Holothuriida at 25.61% and Dendrochirotida at 15.38%. At the family level within Synallactida, Stichopodidae contributes significantly as it accounts for 96.97% of species abundance in this order. Similarly, within Dendrochirotida, Cucumariidae has the highest number of mitogenomes providing an abundance rate of 83.33%. The genera *Apostichopus* and *Stichopus*, belonging to the Synallactida order, play a pivotal role in providing substantial support (84.85%) to this taxonomic group. Similarly, the genera *Holothuria* and *Actinopyga* serve as the cornerstone in supporting a significant proportion (95.00%) of the Holothuriida order.

The violet-red sea cucumber *Benthodytes* sp. Gxx-2023, belonging to the genus *Benthodytes* in the family Psychropotidae, order Elasipodida, class Holothuridae, possesses a mitochondrial genome sequence size of 17,386 bp. It comprises 13 PCGs and 24 non-coding RNAs (2 rRNA genes and 22 tRNA genes), exhibiting similarity to known mitochondrial genomes of other sea cucumbers. Furthermore, it possesses two distinct control regions, measuring 882 bp and 1,153 bp, respectively, which distinguishes it from other sea cucumbers that typically have only one control region. The AT content was 65.33%, indicating a preference for AT base pairs similar to other Holothuroidea and Echinoidea species [19, 20]. It is well-established that the base composition of animal mitochondrial genomes exhibits non-random patterns, with AT-skew and GC-skew values reflecting biases in base composition[19]. Most known sea cucumbers within the class Holothuridae exhibit positive AT-skew values, implying a higher occurrence of adenine (A) than thymine (T), possibly due to shared environmental stresses during evolution that impact mitochondrial DNA transcription or replication [21, 22]. In contrast, our study reveals a negative AT-skew value (-0.07603) for *Benthodytes* sp. Gxx-2023, highlighting its divergence from most sea cucumbers within the Holothuridae class. Additionally, *B. marianensis* (-0.066) and *Scotoplanes* sp. H8 (-0.060), both inhabiting the western Pacific Ocean (*B. marianensis*: 11˚47.9757’ N, 142˚6.8535’E, 5,556 m depth; *Scotoplanes* sp. H8: 39°18′20″-19′″N, 142°49′48″54″E, 1,672–1,692 m depth), also display negative AT-skew values potentially linked to environmental factors and evolutionary mechanisms. Notably, *Benthodytes* sp. Gxx-2023 represents the most significant AT bias observed among known sea cucumber species to date. The distinctiveness of this phenomenon arises from the robust association between the second position of the codon and the control region, with AT-skew values of -0.15715 and -0.15491, respectively. However, rRNA counteracts this bias due to its positive AT-skew value (0.033009).

Mitochondrial PCGs in Gxx-2023, like other sea cucumbers, generally have 13 genes, which is relatively conservative [23–27]. Among the 13 PCGs, there are 11 electron transport system genes (complex I, Complex IV, *cytb*) and 2 oxidative phosphorylation genes (*atp6*, *atp8*). Complex I consists of seven subunits of reductase complexes (*nad4l*, *nad1*, *nad2*, *nad3*, *nad4*, *nad5* and *nad6*), while Complex IV includes three subunits of oxidase (*cox1*, *cox2*, and *cox3*). In general, the homology of mitochondrial genes and encoded proteins of *Benthodytes* sp. Gxx-2023 is relatively low compared to other sea cucumbers, with only around 90% and 97% homology to the genes and proteins of its congeneric species *B. marianensis*, respectively. Furthermore, when compared to other sea cucumbers, the homology is exceptionally low. As expected, COX1 exhibits the highest homology, which is also the key basis for its significance in evolutionary analysis of sea cucumbers. The types of mitochondrial genes of Gxx-2023, are relatively conservative and perform consistent functions, primarily playing an important role in mitochondrial energy production and conversion. For instance, both ATP6 and ATP8 act as subunits of the F0 component of the ATP synthase complex located on the mitochondrial membrane. This complex is responsible for synthesizing ATP from ADP by utilizing a proton gradient established through electron transport complexes within the respiratory chain. Together, the three subunits, COX1, COX2 and COX3, constitute the oxidase enzyme complex which plays a crucial role in mitochondrial energy production and conversion. Moreover, COX3 facilitates the coupling of reduced quinones' oxidation with molecular oxygen's reduction to water while simultaneously pumping protons to establish a proton gradient utilized for ATP synthesis. This pathway is vital for energy metabolism and electron transport [28]. NAD4L, NAD1, NAD2, NAD3, NAD4, NAD5, and NAD6 collectively form several subunits of the NADH dehydrogenase enzyme, which catalyzes the transfer of two electrons from NADH to ubiquinone in a reaction that is linked to proton translocation across the membrane [29, 30]. Although the proteins of Gxx-2023 shares similar functions with other sea cucumbers, its low sequence homology results in unique structural domains, which may be reflected in its substrate binding ability or catalytic efficiency. KEGG annotation analysis reveals the involvement of mitochondrial gene Gxx-2023 in sea cucumber's Energy metabolism, Signal transduction, Circulatory system, Environmental adaptation, Processing Signal transduction [31]. The GO functional analysis highlights its significance in various biological processes [32]. Furthermore, eggNOG analysis suggests that mitochondrial genes play a crucial role in energy production and conversion within mitochondria [33].

The *Benthodytes* sp. Gxx-2023 mitochondrial genome consists of 61 codons exhibiting distinct usage preferences with other sea cucumbers. This is evident from the frequency at which each codon is utilized (Ile > Leu-CUN > Phe > Leu-UUR > Ser-UCN), as well as the utilization of synonymous codons (Ser > Leu > Val > Gln > Ser). Notably, these preferences differ from those observed in other sea cucumbers, thereby highlighting its unique characteristics. The 13 genes have only two start codons, namely ATG and ATT. Among these genes, 12 initiate with ATG as the start codon, while *nad4l* is the sole gene that begins with ATT. It should be noted that reports indicate the usage of GTG as a start codon in some sea cucumber mitochondrial genomes (e.g., *A. japonicus nad1* starts with GTG), which differs from Gxx-2023. The stop codons of certain sea cucumber mitogenome genes remain intact. In Gxx-2023, 10 genes exhibit complete codon forms, including *cox1* with TAG serving as the stop codon. Nine genes (*atp6*, *atp8*, *nd4l*, *nad1*, *nad2*, *nad3*, *nad5*, *nad6* and *cox3*) utilize TAA as their respective stop codons. However, the remaining three genes (*cox2*, *nad4* and *cytb*) possess incomplete stop codons consisting only of a single "T", which may potentially form a "TAA" stop codon after mRNA transcription to terminate the translation process [34, 35]. The study revealed significant disparities in the codon usage proportions among deep-sea and shallow sea cucumbers. For instance, the four most frequently utilized codons in deep-sea cucumbers are TTT (Phe), TTA (Leu), ATT (Ile), and ATA (Met), all of which consist of A and T bases. Differently, certain shallow sea cucumbers predominantly employ TTC (Phe), TTA (Leu), CTA (Leu), and ATA (Met) codons, albeit with a minor presence of C bases [19]. The commonly used codons within the 13 PCGs of *Benthodytes* sp. Gxx-2023 also exhibit an AT bias with a content of 66.11%, similar with other sea cucumbers [22]. Generally, the Third position's AT content tends to be higher than that of the First and Second positions in sea cucumbers as well as other organisms like Echinoidea, abalone, oyster etcetera. However, for *Benthodytes* sp. Gxx-2023 specifically, its second position displays a higher AT content (66.56%) compared to both the first position (65.43%) and third position (66.35%), possibly due to natural selection factors influencing this phenomenon as observed similarly in *A. japonicus* (GenBank No.FJ906623) where its second AT content (65.l%) surpasses that of its first (57.4%) and third (61.8%) positions too. Of course, there are also few sea cucumbers with high AT content in the first codon, such as *B. argus*, whose AT content is 59.32, 59.11 and 58.23% in order [36]. The AT content varies among the three sites; however, their codons exhibit conservatism with a predominant occurrence of AT endings. Consequently, it can be inferred that the evolution of codon usage in sea cucumbers may be attributed to their adaptive response towards survival in low-nutrient food resources [37].

Animal mitochondrial gene rearrangement is classified into four types: inversion, translocation, reverse transposition, and tandem duplication-random losses (TDRL) [38]. Our findings indicate that sea cucumbers primarily undergo transpositions (in every order) and reverse transpositions (specifically in Apodida). Furthermore, compared to *Benthodytes* and *Cucumaria*, other sea cucumbers experience tandem duplication-random losses. In general, the sea cucumber species within Holothuriida and some within Synallactida exhibit a fundamental resemblance to the echinoderm ground pattern, which is subsequently followed by Phyllophoridae in Dendrochirotida and Elpidiidae in Elasipodida. The tRNA genes are observed to be the most mobile elements within the mitogenome, appearing in all PCGs, tRNA and rRNA genes. Among these, Apodida demonstrates the highest degree of mitochondrial gene rearrangement, followed by *Cucumaria* and *Benthodytes*. The gene arrangement of *Benthodytes* sp. Gxx-2023 aligns with that of *B. marianensis* but differs from other species within the same order. TDRL-induced rearrangements also impact intergenic distances enabling *Benthodytes* sp. Gxx-2023 to acquire a larger mtDNA size which may be related to environmental changes [39].

Evolutionary analysis revealed a close relationship between *Benthodytes* Gxx.2023 and *B. marianensis*, confirming the classification of this sea cucumber within the genus *Benthodytes* and supporting its monophyly. The genus *Benthodytes* belongs to the family Psychropotidae and is classified under the order Elasipodida, along with the family Elpidiidae as sister groups. The order Dendrochirotida, Synallactida and Holothuriid form separate branches that are closely related to the order Elasipodida, consistent with previous studies [40]. However, Apodida shows distant relationships with all aforementioned orders. A more comprehensive analysis of mitochondrial genomes in sea cucumbers will contribute to a better understanding of their phylogenetic relationships. Nevertheless, limited data on sea cucumber mitochondrial genomes currently exists; thus, it is necessary to obtain complete mitochondrial genomes from additional species and conduct comprehensive studies in order to further elucidate phylogenetic relationships within sea cucumbers at a molecular level and identify potential cryptic species.

## Conclusions

We have successfully sequenced, assembled, annotated, and analyzed the complete mitochondrial genome of *Benthodytes* sp. Gxx-2023 residing in the western Pacific Ocean. The entire mitogenome spans a total length of 17,386 bp and exhibits a conserved gene composition comprising 37 genes (13 PCGs, 2 rRNA genes, and 22 tRNA genes). However, it demonstrates limited homology in terms of gene sequence with other sea cucumber species and showcases distinct rearrangements within its mitogenome during evolutionary processes. This study significantly contributes to the understanding of sea cucumber diversification, particularly Elasipodida, thereby holding immense implications for advancing sea cucumber genomics research and exploring novel resources related to this marine organism.

### Supplementary Information

Supplementary Material 1: Figure S1. Sequencing depth and coverage map of *Benthodytes* sp. Gxx-2023 mitogenome.

Supplementary Material 2: Table S1. Detailed information of mitochondrial genome in Holothurioidea.

Supplementary Material 3: Table S2. Homology comparison analysis of *Benthodytes* sp. Gxx-2023 mitochondrial genes.

Supplementary Material 4: Table S3. Homology comparison analysis of *Benthodytes* sp. Gxx-2023 mitochondrial proteins.

Supplementary Material 5: Table S4. GO functional analysis of *Benthodytes* sp. Gxx-2023 mitogenome genes.

Supplementary Material 6: Table S5. KEGG category analysis of *Benthodytes* sp. Gxx-2023 mitogenome genes.

## Data Availability

The sequence and annotation of *Benthodytes* sp. Gxx-2023 mtDNA were submitted to the NCBI, with the accession number OR992091 in GenBank, which was released on 10-JAN-2024.

## Abbreviations

atp6: ATP synthase F0 subunit 6
atp8: ATP synthase F0 subunit 8
BLAST: Basic Local Alignment Search Tool
cox1: Cytochrome c oxidase subunit 1
cox2: Cytochrome c oxidase subunit 2
cox3: Cytochrome c oxidase subunit 3
cytb: Cytochrome b
gDNA: Genomic DNA
mtDNA: Mitochondrial DNA
nad1: NADH dehydrogenase subunit 1
nad2: NADH dehydrogenase subunit 2
nad3: NADH dehydrogenase subunit 3
nad4: NADH dehydrogenase subunit 4
nad5: NADH dehydrogenase subunit 5
nad6: NADH dehydrogenase subunit 6
nad4l: NADH-ubiquinone/plastoquinone oxidoreductase, chain 4L
NCBI: National Center for Biotechnology Information
PCGs: Protein-coding genes
RSCU: Relative synonymous codon usage
TDRL: Tandem duplication-random losses
