# Supplementary material for: A novel deep-benthic sea cucumber species of Benthodytes (Holothuroidea, Elasipodida, Psychropotidae) and its comprehensive mitochondrial genome sequencing and evolutionary analysis
Source: BMC Genomics. 2024 Jul 13;25:689. doi: 10.1186/s12864-024-10607-5 (PMC11245801; doi:10.1186/s12864-024-10607-5)
Supplement: Supplementary file 1 — Supplementary Material 1: Figure S1. Sequencing depth and coverage map of Benthodytes sp. Gxx-2023 mitogenome. [file 12864_2024_10607_MOESM1_ESM.pdf]

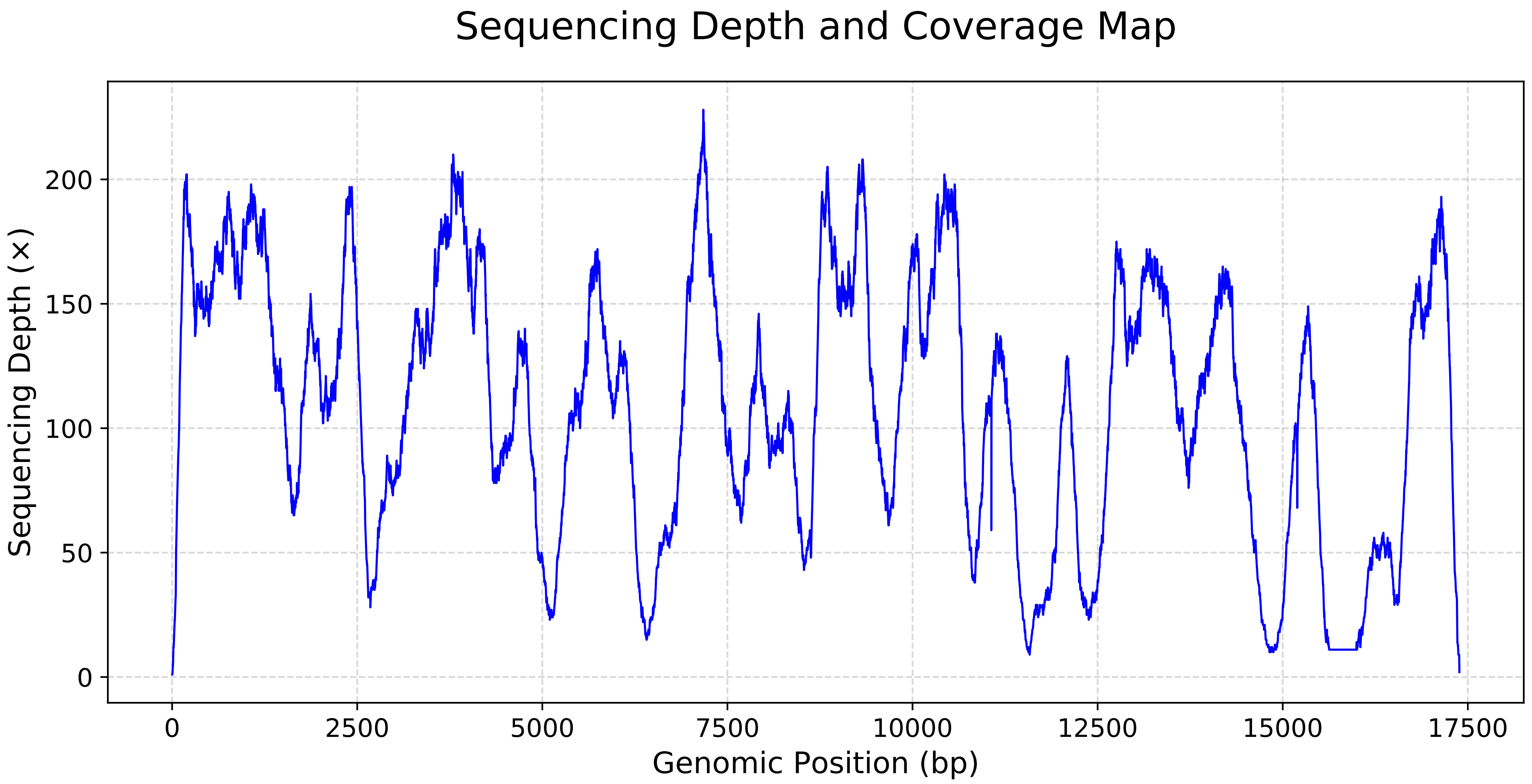

(1) Total genome length = 17,386 bp
(2) Average depth = 110.91 ×
(3) Maximal depth = 228 ×
(4) Minimal depth = 1 ×
