# Supplementary material for: A novel deep-benthic sea cucumber species of Benthodytes (Holothuroidea, Elasipodida, Psychropotidae) and its comprehensive mitochondrial genome sequencing and evolutionary analysis
Source: BMC Genomics. 2024 Jul 13;25:689. doi: 10.1186/s12864-024-10607-5 (PMC11245801; doi:10.1186/s12864-024-10607-5)
Supplement: Supplementary file 2 — Supplementary Material 2: Table S1. Detailed information of mitochondrial genome in Holothurioidea. [file 12864_2024_10607_MOESM2_ESM.docx]

Table S1 Detailed information of mitochondrial genome in Holothurioidea

| NO. | Order | Family | Species | Sizes  (bp) | Complete  ? | Accession No. |
| --- | --- | --- | --- | --- | --- | --- |
|  | Elasipodida | Psychropotidae | *Benthodytes* sp. Gxx-2023 | 17,386 | √ | OR992091.1 |
|  | Elasipodida | Psychropotidae | *Benthodytes marianensis* | 17,567 | √ | MH208310.1 |
|  | Elasipodida | Elpidiidae | *Peniagone* sp. YYH-2013 | 15,507 | √ | KF915304.1 |
|  | Elasipodida | Elpidiidae | *Scotoplanes* sp. H5 | 15,501 | × | LC416625.1 |
|  | Elasipodida | Elpidiidae | *Scotoplanes* sp. TT-2017 | 15,913 | √ | LC416624.1 |
|  | Elasipodida | Elpidiidae | *Scotoplanes* sp. H8 | 15,910 | √ | LC416626.1 |
|  | Synallactida | Stichopodidae | *Parastichopus californicus* | 16,727 | √ | KP398509.1 |
|  | Synallactida | Stichopodidae | *Parastichopus nigripunctatus* | 16,112 | √ | AB525762.1 |
|  | Synallactida | Stichopodidae | *Isostichopus badionotus* | 16,318 | √ | MZ188901.1 |
|  | Synallactida | Stichopodidae | *Stichopus horrens* | 16,315 | √ | MN128376.1 |
|  | Synallactida | Stichopodidae | *Stichopus horrens* isolate Hainan province | 16,261 | √ | MZ321056.1 |
|  | Synallactida | Stichopodidae | *Stichopus horrens* | 16,257 | √ | HQ000092.1 |
|  | Synallactida | Stichopodidae | *Stichopus monotuberculatus* | 16,274 | √ | MN276189.1 |
|  | Synallactida | Stichopodidae | *Stichopus ocellatus* | 16,260 | √ | MZ292734.1 |
|  | Synallactida | Stichopodidae | *Stichopus* sp. SF-2010 | 16,257 | √ | HM853683.2 |
|  | Synallactida | Stichopodidae | *Stichopus chloronotus* | 16,249 | √ | MZ052220.1 |
|  | Synallactida | Stichopodidae | *Stichopus chloronotus* strain lv | 16,247 | √ | MW218897.1 |
|  | Synallactida | Stichopodidae | *Stichopus naso* | 16,239 | √ | MZ469138.1 |
|  | Synallactida | Stichopodidae | *Apostichopus californicus* isolate PC1234 | 16,416 | × | CM036232.1 |
|  | Synallactida | Stichopodidae | *Apostichopus parvimensis* | 16,120 | √ | KU168761.1 |
|  | Synallactida | Stichopodidae | *Apostichopus japonicus* | 16,109 | √ | FJ986223.1 |
|  | Synallactida | Stichopodidae | *Apostichopus japonicus* isolate JBRI-0814-Black | 16,108 | √ | FJ594967.1 |
|  | Synallactida | Stichopodidae | *Apostichopus japonicus* isolate JBRI-0812-Red | 16,107 | √ | FJ594963.1 |
|  | Synallactida | Stichopodidae | *Apostichopus japonicus* isolate red | 16,107 | √ | MK216561.1 |
|  | Synallactida | Stichopodidae | *Apostichopus japonicus* isolate LO1 | 16,106 | √ | KP170617.1 |
|  | Synallactida | Stichopodidae | *Apostichopus japonicus* isolate W112 | 16,106 | √ | KP170618.1 |
|  | Synallactida | Stichopodidae | *Apostichopus japonicus* isolate green | 16,106 | √ | MK208925.1 |
|  | Synallactida | Stichopodidae | *Apostichopus japonicus* | 16,105 | √ | AB525437.1 |
|  | Synallactida | Stichopodidae | *Apostichopus japonicus* red type | 16,103 | √ | AB525761.1 |
|  | Synallactida | Stichopodidae | *Apostichopus japonicus* isolate Red race 01 | 16,103 | √ | GU557147.1 |
|  | Synallactida | Stichopodidae | *Apostichopus japonicus* blue type | 16,106 | √ | AB525760.1 |
|  | Synallactida | Stichopodidae | *Apostichopus japonicus* isolate Red race 02 | 16,103 | √ | GU557148.1 |
|  | Synallactida | Stichopodidae | *Apostichopus japonicus* isolate JBRI-0813-Green | 16,102 | √ | FJ594968.1 |
|  | Synallactida | Stichopodidae | *Apostichopus japonicus* isolate H321 | 16,100 | √ | KP170616.1 |
|  | Synallactida | Stichopodidae | *Apostichopus japonicus* | 16,099 | √ | FJ906623.1 |
|  | Synallactida | Stichopodidae | *Apostichopus japonicus* | 16,096 | √ | EU294194.1 |
|  | Synallactida | Stichopodidae | *Apostichopus japonicus* isolate black | 16,096 | √ | MK216560.1 |
|  | Synallactida | Stichopodidae | *Thelenota ananas* | 15,858 | √ | MW548268.1 |
|  | Synallactida | Synallactidae | *Synallactes* sp. Y30071 | 15,920 | × | MT559281.1 |
|  | Holothuriida | Holothuriidae | *Holothuria leucospilota* voucher HL2 | 15,907 | × | ON584426.1 |
|  | Holothuriida | Holothuriidae | *Holothuria leucospilota* | 15,906 | × | MK801674.1 |
|  | Holothuriida | Holothuriidae | *Holothuria leucospilota* | 15,904 | √ | MN594790.1 |
|  | Holothuriida | Holothuriidae | *Holothuria leucospilota* | 15,839 | √ | MN276190.1 |
|  | Holothuriida | Holothuriidae | *Holothuria fuscocinerea* | 15,890 | × | MK391177.1 |
|  | Holothuriida | Holothuriidae | *Holothuria forskali* | 15,841 | √ | FN562582.1 |
|  | Holothuriida | Holothuriidae | *Holothuria spinifera* strain huang | 15,835 | √ | MW218896.1 |
|  | Holothuriida | Holothuriidae | *Holothuria spinifera* strain S-4 | 15,812 | √ | MN816440.1 |
|  | Holothuriida | Holothuriidae | *Holothuria fuscocinerea* | 15,827 | √ | MN542416.1 |
|  | Holothuriida | Holothuriidae | *Holothuria pervicax* | 15,790 | √ | MK328500.1 |
|  | Holothuriida | Holothuriidae | *Holothuria scabra* | 15,779 | √ | KP257577.1 |
|  | Holothuriida | Holothuriidae | *Holothuria leucospilota* | 15,904 | √ | MK940237.1 |
|  | Holothuriida | Holothuriidae | *Holothuria hilla* | 15,744 | √ | MN163001.1 |
|  | Holothuriida | Holothuriidae | *Holothuria edulis* | 15,743 | √ | MT084774.1 |
|  | Holothuriida | Holothuriidae | *Holothuria poli* | 15,907 | √ | LR694133.1 |
|  | Holothuriida | Holothuriidae | *Holothuria fuscogilva* | 15,633 | √ | MZ305460.1 |
|  | Holothuriida | Holothuriidae | *Bohadschia argus* | 15,656 | √ | OL741685.1 |
|  | Holothuriida | Holothuriidae | *Actinopyga echinites* | 15,619 | √ | MN793975.1 |
|  | Holothuriida | Holothuriidae | *Actinopyga lecanora* strain hui | 15,569 | √ | MW218894.1 |
|  | Holothuriida | Holothuriidae | *Actinopyga lecanora* | 15,568 | √ | [MW248463.1](https://www.ncbi.nlm.nih.gov/nuccore/MW248463.1) |
|  | Dendrochirotida | Cucumariidae | *Cucumaria miniata* | 17,538 | √ | AY182376.1 |
|  | Dendrochirotida | Cucumariidae | *Cucumaria frondosa* | 17,507 | √ | MZ321055.1 |
|  | Dendrochirotida | Cucumariidae | *Colochirus quadrangularis* strain haishen | 17,277 | √ | MW218895.1 |
|  | Dendrochirotida | Cucumariidae | *Colochirus quadrangularis* | 17,157 | √ | MT108721.1 |
|  | Dendrochirotida | Cucumariidae | *Colochirus robustus* | 17,171 | × | MN966676.1 |
|  | Dendrochirotida | Cucumariidae | *Thyonella gemmata* voucher UF:021831 | 15,696 | √ | MZ463652.1 |
|  | Dendrochirotida | Cucumariidae | *Ocnus glacialis* | 16,778 | √ | OR420052.1 |
|  | Dendrochirotida | Cucumariidae | *Cercodemas anceps* | 16,539 | √ | MW044622.1· |
|  | Dendrochirotida | Cucumariidae | *Neocucumis proteus* | 16,495 | √ | MZ305458.1 |
|  | Dendrochirotida | Cucumariidae | *Pseudocolochirus violaceus* | 15,756 | √ | MT587564.1 |
|  | Dendrochirotida | Phyllophoridae | *Phyllophorella liuwutiensis* | 15,969 | √ | MN198190.1 |
|  | Dendrochirotida | Phyllophoridae | *Phyrella fragilis* | 15,910 | √ | MZ305459.1 |
|  | Apodida | Chiridotidae | *Chiridota heheva* | 17,200 | √ | MW357261.1 |
|  | Apodida | Chiridotidae | *Chiridota* sp. SS-2021 | 17,199 | √ | MW357262.1 |
|  | Apodida | Chiridotidae | *Chiridotidae* sp. KJ-Belize-E1_1 | 16,880 | × | MT877116.1 |
|  | Apodida | Synaptidae | *Protankyra verrilli* | 16,970 | × | ON018239.1 |
|  | Apodida | Synaptidae | *Euapta godeffroyi* | 16,410 | √ | LC704718.1 |
|  | Molpadida | Caudinidae | *Acaudina molpadioides* | 16,028 | × | MK050109.1 |
|  | UNVERIFIED | Holothuroidea | *Holothuroidea* sp. FZ-2017 | 15,852 | √ | MF667551.1 |
