# Supplementary material for: A novel deep-benthic sea cucumber species of Benthodytes (Holothuroidea, Elasipodida, Psychropotidae) and its comprehensive mitochondrial genome sequencing and evolutionary analysis
Source: BMC Genomics. 2024 Jul 13;25:689. doi: 10.1186/s12864-024-10607-5 (PMC11245801; doi:10.1186/s12864-024-10607-5)
Supplement: Supplementary file 5 — Supplementary Material 5: Table S4. GO functional analysis of Benthodytes sp. Gxx-2023 mitogenome genes. [file 12864_2024_10607_MOESM5_ESM.docx]

Table S4 GO functional analysis of *Benthodytes* sp. Gxx-2023 mitogenome genes

| NO. | Namespace | Description | Number | Percent | ID | Genes |
| --- | --- | --- | --- | --- | --- | --- |
| 1 | biological_process | reproduction | 1 | 0.0909 | GO:0000003 | *cob* |
| 2 | molecular_function | catalytic activity | 8 | 0.7272 | GO:0003824 | *atp6;cob;cox1;nad1;nad2;nad3;nad4;nad5* |
| 3 | molecular_function | transporter activity | 2 | 0.1818 | GO:0005215 | *atp6;cox1* |
| 4 | molecular_function | binding | 2 | 0.1818 | GO:0005488 | *cob;nad2* |
| 5 | cellular_component | intracellular | 11 | 1 | GO:0005622 | *atp6;cob;cox1;cox2;cox3;nad1;nad2;nad3;nad4;nad5;nad6* |
| 6 | biological_process | metabolic process | 9 | 0.8181 | GO:0008152 | *atp6;cob;cox1;cox2;nad1;nad2;nad3;nad4;nad5* |
| 7 | biological_process | cellular process | 11 | 1 | GO:0009987 | *atp6;cob;cox1;cox2;cox3;nad1;nad2;nad3;nad4;nad5;nad6* |
| 8 | biological_process | reproductive process | 1 | 0.0909 | GO:0022414 | *cob* |
| 9 | biological_process | multicellular organismal process | 4 | 0.3636 | GO:0032501 | *atp6;cob;cox1;nad4* |
| 10 | biological_process | developmental process | 4 | 0.3636 | GO:0032502 | *atp6;cob;cox1;nad4* |
| 11 | cellular_component | protein-containing complex | 10 | 0.909 | GO:0032991 | *atp6;cob;cox1;cox2;cox3;nad1;nad2;nad3;nad4;nad5* |
| 12 | biological_process | locomotion | 1 | 0.0909 | GO:0040011 | *atp6* |
| 13 | biological_process | response to stimulus | 7 | 0.6363 | GO:0050896 | *cob;cox1;nad1;nad3;nad4;nad5;nad6* |
| 14 | biological_process | localization | 3 | 0.2727 | GO:0051179 | *atp6;cob;cox1* |
| 15 | biological_process | multi-organism process | 1 | 0.0909 | GO:0051704 | *cob* |
| 16 | biological_process | biological regulation | 1 | 0.0909 | GO:0065007 | *atp6* |
| 17 | cellular_component | cellular anatomical entity | 11 | 1 | GO:0110165 | *atp6;cob;cox1;cox2;cox3;nad1;nad2;nad3;nad4;nad5;nad6* |
